# Integrated Multichip Analysis Identifies Potential Key Genes in the Pathogenesis of Nonalcoholic Steatohepatitis

**DOI:** 10.3389/fendo.2020.601745

**Published:** 2020-11-26

**Authors:** Jianzhong Ye, Yishuai Lin, Qing Wang, Yating Li, Yajie Zhao, Lijiang Chen, Qing Wu, Chunquan Xu, Cui Zhou, Yao Sun, Wanchun Ye, Fumao Bai, Tieli Zhou

**Affiliations:** ^1^Department of Clinical Laboratory, The First Affiliated Hospital of Wenzhou Medical University, Wenzhou, China; ^2^School of Laboratory Medicine and Life Sciences, Wenzhou Medical University, Wenzhou, China; ^3^Collaborative Innovation Center for the Diagnosis and Treatment of Infectious Diseases, State Key Laboratory for the Diagnosis and Treatment of Infectious Diseases, The First Affiliated Hospital, School of Medicine, Zhejiang University, Hangzhou, China; ^4^National Clinical Research Center for Infectious Diseases, The First Affiliated Hospital, School of Medicine, Zhejiang University, Hangzhou, China; ^5^Department of Chemotherapy 2, Wenzhou Central Hospital, Wenzhou, China

**Keywords:** nonalcoholic steatohepatitis, hepatic steatosis, microarray, differentially expressed genes, integrated analysis

## Abstract

**Background:**

Nonalcoholic steatohepatitis (NASH) is rapidly becoming a major chronic liver disease worldwide. However, little is known concerning the pathogenesis and progression mechanism of NASH. Our aim here is to identify key genes and elucidate their biological function in the progression from hepatic steatosis to NASH.

**Methods:**

Gene expression datasets containing NASH patients, hepatic steatosis patients, and healthy subjects were downloaded from the Gene Expression Omnibus database, using the R packages biobase and GEOquery. Differentially expressed genes (DEGs) were identified using the R limma package. Functional annotation and enrichment analysis of DEGs were undertaken using the R package ClusterProfile. Protein-protein interaction (PPI) networks were constructed using the STRING database.

**Results:**

Three microarray datasets GSE48452, GSE63067 and GSE89632 were selected. They included 45 NASH patients, 31 hepatic steatosis patients, and 43 healthy subjects. Two up-regulated and 24 down-regulated DEGs were found in both NASH patients vs. healthy controls and in steatosis subjects vs. healthy controls. The most significantly differentially expressed genes were *FOSB* (*P* = 3.43×10^-15^), followed by *CYP7A1* (*P* = 2.87×10^-11^), and *FOS* (*P* = 6.26×10^-11^). Proximal promoter DNA-binding transcription activator activity, RNA polymerase II-specific (*P* = 1.30×10^-5^) was the most significantly enriched functional term in the gene ontology analysis. KEGG pathway enrichment analysis indicated that the MAPK signaling pathway (*P* = 3.11×10^-4^) was significantly enriched.

**Conclusion:**

This study characterized hub genes of the liver transcriptome, which may contribute functionally to NASH progression from hepatic steatosis.

## Introduction

Nonalcoholic steatohepatitis (NASH) is a severe form of nonalcoholic fatty liver disease (NAFLD). Its histological features include simple steatosis, hepatic and systemic inflammation, and liver injury with varying degrees of fibrosis. The global prevalence of NASH is approximately 1.5%–6.5% (1). Patients with NASH, especially those with advanced fibrosis, are more likely to progress to hepatocellular carcinoma (HCC) (2). It has been suggested that NASH will become the principal indication for liver transplantation in the near future (3). NASH is now a major public health concern due to its increasing prevalence and poor prognosis (4).

Historically, the underlying pathogenesis of NASH was explained by the so-called “two-hit” theory (5). The first “hit” was triglyceride accumulation, resulting in hepatic steatosis. The second “hit” includes oxidative stress, hormonal imbalance, and mitochondrial abnormalities, which together contribute to the progression from simple steatosis to NASH. However, accumulated evidence suggests that the “two-hit” theory does not explain many of the multiple molecular and metabolic alterations seen in this disease (6). Moreover, hepatic steatosis is benign and does not progress in most subjects, suggesting NASH may be a heterogeneous disease with a distinct pathogenesis (7). There is thus a pressing need to fully understand the pathogenesis of NASH more completely.

Motivated by this dilemma, our aim was to mine hub genes within the liver transcriptome that can drive progression of hepatic steatosis to NASH, using extant information deposited in the Gene Expression Omnibus (GEO) (8) database at the National Center for Biotechnology Information (NCBI). Datasets that contained NASH patients, hepatic steatosis patients, and healthy subjects were selected from the GEO database, and used to conduct a reliable genome-wide microarray analysis of mRNA expression profiles. First, we identified a set of differentially expressed genes (DEGs) by comparing hepatic steatosis and healthy control samples. Second, we compared the gene expression profile from samples of NASH patients and healthy controls. Third, we pooled those genes that were differentially expressed in both steatosis patients versus healthy controls and in NASH patients versus healthy controls. Finally, 26 DEGs were identified and subjected to additional functional analysis.

## Materials and Methods

### Inclusion and Exclusion Criteria

Initially, we undertook a systematic and comprehensive search within the GEO database (http://www.ncbi.nlm.nih.gov/geo/) (9). Our retrieval strategy used the following search term: {[homo sapiens (Organism)] AND [(non-alcoholic fatty liver disease) OR (nonalcoholic fatty liver disease) OR (NAFLD) OR (steatosis)] AND [(non-alcoholic steatohepatitis) OR (nonalcoholic steatohepatitis) OR (NASH)]}. “Study type” was restricted to “expression profiling by array”. Details of the retrieval strategy and subsequent analysis are presented in **Supplemental Figure 1**.

Datasets meeting the following criteria were included in the study: 1) mRNA expression profiling was conducted using liver samples; 2) GEO datasets contained hepatic steatosis subjects, NASH subjects, and matched healthy controls; 3) definite diagnosis of steatosis or NASH.

Datasets meeting the following criteria were excluded from the study: 1) patients with other diseases, such as hepatocellular carcinoma (HCC); 2) obese subjects matched as controls; 3) ambiguous diagnosis, as such where steatosis and NASH were collectively referred to as NAFLD.

### Data Cleaning

Three datasets: GSE48452 (10), GSE63067 (11), and GSE89632 (12), met all criteria and were included in this study, from 27 studies initially identified by screening GEO. Raw microarray data were downloaded using R studio (https://www.rstudio.com/) with biobase and GEOquery packages. The data were then annotated and merged, using a custom-written Perl script (https://www.perl.org/). All probes were mapped to their corresponding Entrez Gene ID. When multiple probes matched with one gene, their mean expression was used. Probes were excluded if they did not map to any known genes. The microarray mRNA expression data were then batch normalized using R packages sva and limma. Obese patients (n=16) or individuals having had bariatric surgery (n=19) in GSE48452 were excluded, based on the predefined exclusion criteria above. Details of included datasets are outlined in **Table 1**.

**Table 1:** Characteristics of the included GEO datasets.

GSE ID	Participants included	Tissues	Analysis type	Platform	Year
GSE48452	H = 12, S = 9, N = 17	Liver	Array	GPL11532	2013
GSE63067	H = 7, S = 2, N = 9	Liver	Array	GPL570	2014
GSE89632	H = 24, S = 20, N = 19	Liver	Array	GPL14951	2016
Total:	H = 43, S = 31, N = 45				

H, healthy control; S, steatosis; N, NASH.

### Identification of Differentially Expressed Genes

Pre-processed mRNA expression data were analyzed to identify DEGs using the R package limma. The resulting *P*-values were corrected using the Benjamini and Hochberg approach, at a False Discovery Rate (FDR) of 5%. Genes with an adjusted *P*-value of <0.05 and log fold change (FC) greater than 1 were considered as DEGs. The log FC is the logarithm of the ratio of the change in expression for each gene between groups (13). Liver transcriptome profiles from steatosis and NASH samples were first compared to healthy controls. Data were then pooled using principal component analysis (PCA) with the prcomp function of R package stats, and visualized using R packages ggplot2. PCA is an orthogonal linear transformation of an existing coordinate frame such that the largest variance of the projected data lies on the first coordinate, or so-called first principal component (PC1), the second largest variance on the second coordinate, or PC2, which is perpendicular to PC1, and so on (14). Volcano plots were used to visualize the differential gene expression between the groups. Venn diagrams were plotted to identify DEGs present in both steatosis patients vs. healthy control comparisons and comparisons of NASH subjects vs. healthy controls. Heatmaps visualized the expression levels of the commonly shared DEGs in NASH patients and healthy subjects, and were plotted based on hierarchical clustering analysis using the R package pheatmap. The expression levels of the top 10 DEGs in NASH patients and healthy controls were shown as box plots, using the R package ggplot2.

### Functional Annotation and Enrichment Analysis of DEGs

To understand the biological function and signaling pathways of the commonly shared DEGs involved in NASH, the 26 identified DEGs were subjected to enrichment analysis within the Gene Ontology (GO; http://www.geneontology.org/) database and pathway analysis within the Kyoto Encyclopedia of Genes and Genomes (KEGG; https://www.kegg.jp/) using the R package ClusterProfile. Adjusted *P-*values of <0.05 and *Q-*values <0.05 were used to define the working threshold for statistical significance (15, 16). Terms included in the GO enrichment analysis were: “biological process” (BP), “cellular component” (CC), and “molecular function” (MF).

### Construction of the Protein–Protein Interaction Network

The Search Tool for the Retrieval of Interacting Genes database (STRING version 11.0; https://string-db.org/) (17) was used to construct protein-protein interaction (PPI) networks for the 26 identified DEGs. A combined score above 0.4 was used as the selection threshold. Then, Cytoscape software (http://www.cytoscape.org) (18) was used to visualize the PPI networks.

### Expression Validation of DEGs

Validation of expression was undertaken using data from a high throughput sequencing GEO dataset (GSE126848: 15 steatosis patients, 16 NASH patients, and 14 healthy controls). A count-based differential expression analysis of RNA-seq data was undertaken using the edgeR function (19). A *P*-value of <0.05 was set as the significance threshold (20).

## Results

### Identification and Analysis of 26 DEGs Present in Both NASH vs. Healthy Controls and Steatosis vs. Healthy Controls

**Figure 1:**
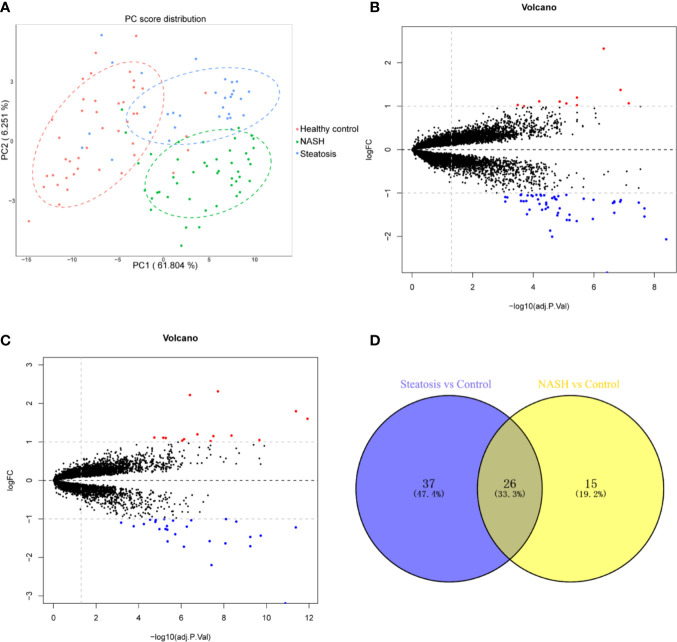
Identification of differentially expressed genes (DEGs) in both NASH and steatosis subjects when compared to healthy controls. **(A)** Principal component analysis (PCA) showing that steatosis patients, NASH patients, and healthy subjects are clearly separated into distinct clusters. **(B)** Volcano plot showing 10 up-regulated genes (red dots) and 53 down-regulated genes (blue dots) in steatosis patients when compared to healthy controls; log fold change (FC) >1, *P <*0.05. **(C)** Volcano plot showing 14 up-regulated genes (red dots) and 27 down-regulated (blue dots) in NASH patients when compared to healthy controls; log fold change (FC) >1, *P <*0.05. **(D)** Venn diagram displaying 26 DEGs present in both NASH and steatosis subjects.

**Figure 2:**
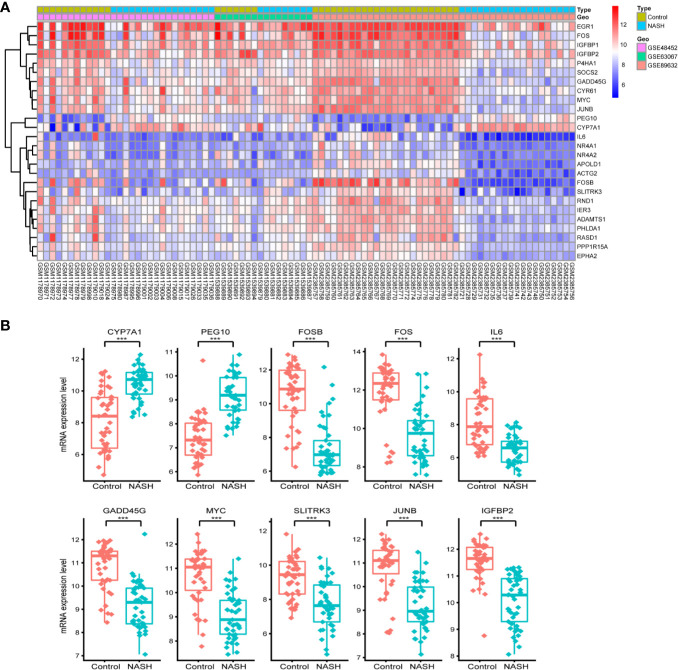
Characterizing the 26 differentially expressed genes (DEGs) shared by NASH and steatosis subjects. **(A)** A heatmap of the 26 DEGs. Each row represents a gene and each column represents a sample. The color scale on the right illustrates the relative expression level of DEGs from blue (low) to red (high). **(B)** Scatter plot of expression levels of the identified top 10 DEGs. The top two up-regulated genes (CYP7A1, PEG10) and the top eight down-regulated genes (FOSB, FOS, IL6, GADD45G, MYC, SLITRK3, JUNB, IGFBP2) are ranked by their respective change in expression level. Detailed information on 26 genes is listed in **Table 2**. ***P < 0.001.

First, we merged and normalized three GEO datasets: GSE48452, GSE63067, and GSE89632. DEGs were identified between steatosis patients and healthy controls and between NASH patients and healthy controls. A PCA analysis indicated the presence of three distinct clusters (**Figure 1A**). 63 genes were differentially expressed between steatosis and healthy controls: 10 up-regulated and 53 down-regulated (**Figure 1B**, **Supplemental Table 1**). 41 genes were differentially expressed between NASH and healthy controls: 14 up-regulated and 27 down-regulated (**Figure 1C**, **Supplemental Table 2**). By using a Venn diagram, 26 DEGs were found to be common to both analyses: 2 up-regulated and 24 down-regulated (**Figure 1D**, **Supplemental Table 3**). A heatmap indicated distinct expression patterns exhibited by the 26 DEGs, when comparing NASH patients and healthy subjects (**Figure 2A**). This suggests these genes may play some role in driving NASH progression. **Table 2** lists the top 10 most statistically significant DEGS in NASH, ordered by the magnitude of altered expression. The 10 DEGs included two up-regulated genes (*CYP7A1*, and *PEG10*), and eight down-regulated genes (*FOSB*, *FOS*, *IL6*, *GADD45G*, *MYC*, *SLITRK3*, *JUNB*, *IGFBP2*). Box plots of mRNA expression levels of the 10 DEGs are shown in **Figure 2B**.

**Table 2:** Top 10 aberrantly expressed DEGs in NASH.

Gene symbol	Gene	Log FC	*P*-value	Adjusted *P*-value
*CYP7A1*	Cytochrome P450 family 7 subfamily A member 1	2.31	2.87E−11	1.89E−08
*PEG10*	Paternally expressed 10	1.80	5.42E−16	4.11E−12
*FOSB*	FosB proto-oncogene	−3.19	3.43E−15	1.30E−11
*FOS*	Fos proto-oncogene	−2.20	6.26E−11	3.80E−08
*IL6*	Interleukin 6	−1.72	2.71E−09	7.47E−07
*GADD45G*	Growth arrest and DNA damage inducible gamma	−1.71	4.52E−13	5.71E−10
*MYC*	MYC proto-oncogene	−1.64	1.11E−11	8.45E−09
*SLITRK3*	SLIT and NTRK like family member 3	−1.58	3.22E−08	4.40E−06
*JUNB*	JunB proto-oncogene	−1.58	9.20E−11	4.65E−08
*IGFBP2*	Insulin like growth factor binding protein 2	−1.47	4.17E−13	5.71E−10

### Significant Biological Differences of the 26 DEGs as Carried Out by Gene Ontology Enrichment Analysis

**Figure 3:**
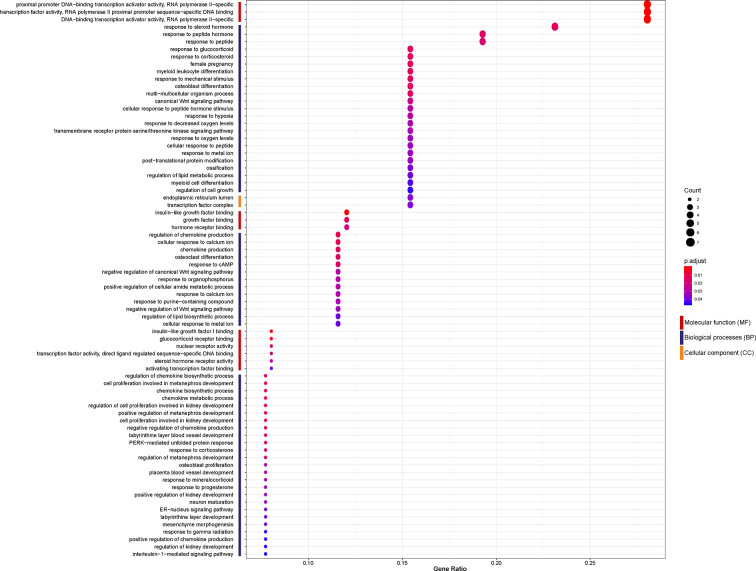
Enriched gene ontology (GO) functions of the 26 differentially expressed genes (DEGs) according to three complementary biological roles: molecular function (MF), biological process (BP), and cellular component (CC).

To investigate the biological function of the 26 identified DEGs shared between NASH and steatosis, a gene ontology (GO) enrichment analysis was undertaken (**Figure 3**). The most significantly enriched GO term of molecular function (MF) was proximal promoter DNA−binding transcription activator activity, RNA polymerase II−specific (GO:0001077; *P* = 1.30×10^-5^). Response to steroid hormone was the most significantly enriched GO term of biological processes (BP) (GO:0048545; *P* = 1.36×10^-5^). In terms of cellular component (CC) ontology, endoplasmic reticulum lumen was significantly enriched (GO:0005788; *P* = 6.17×10^-4^).

### A Signature of Signaling Pathways of the 26 DEGs That Were Revealed by Kyoto Encyclopedia of Genes and Genomes Pathway Analysis

**Figure 4:**
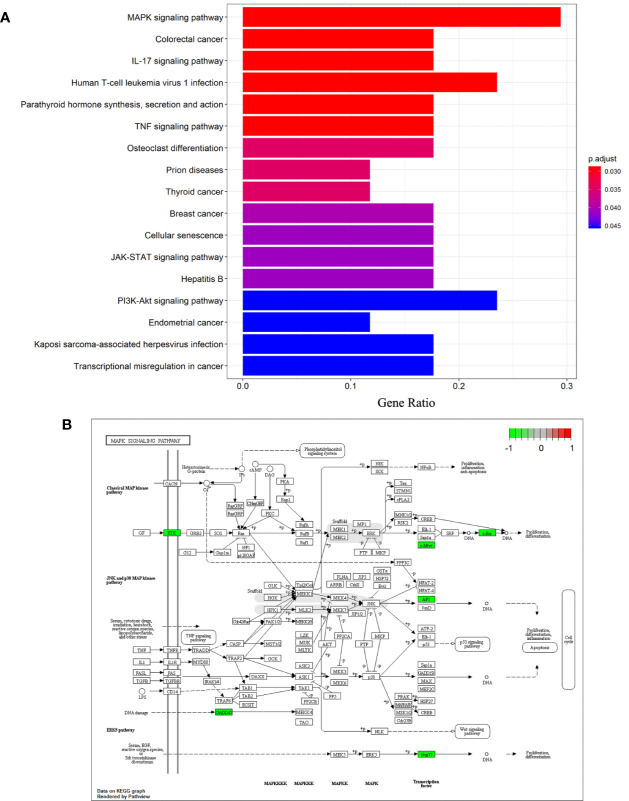
Differentiation-associated KEGG functional analysis. **(A)** A histogram of KEGG biological signaling pathways. **(B)** Colored map of the MAPK signaling pathway.

Analysis of Kyoto Encyclopedia of Genes and Genomes (KEGG) pathways was undertaken to elucidate the signaling pathways of the 26 DEGs identified previously. “MAPK signaling pathway”, “Colorectal cancer”, “IL-17 signaling pathway”, “Human T-cell leukemia virus 1 infection”, “Parathyroid hormone synthesis, secretion and action”, “TNF signaling pathway”, “Osteoclast differentiation”, “Prion diseases”, “Thyroid cancer”, “Breast cancer”, “Cellular senescence”, “JAK-STAT signaling pathway”, “Hepatitis B”, “PI3K-Akt signaling pathway”, “Endometrial cancer”, “Kaposi sarcoma-associated herpesvirus infection”, “Transcriptional misregulation in cancer” were significantly enriched KEGG pathways. A histogram indicating the percentage of genes affected in these pathways is shown in **Figure 4A**. The MAPK signaling pathway was found to be most significantly enriched in NASH patients (hsa04010; *P* = 3.11×10^-4^), and is shown in **Figure 4B**.

### Key Candidate Genes Were Identified With DEGs Protein–Protein Interaction Network

**Figure 5:**
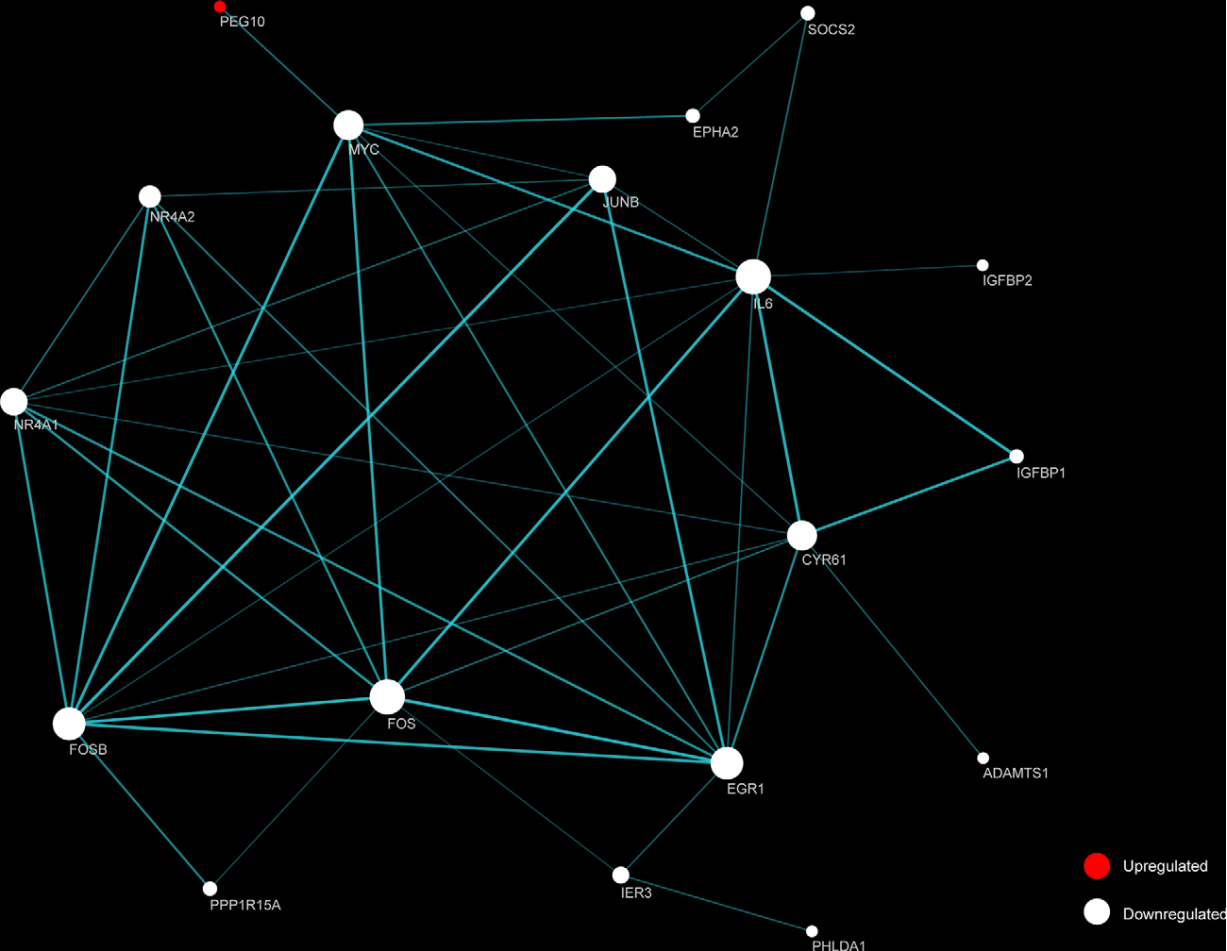
Functional protein-protein interaction (PPI) network analysis of the differentially expressed genes (DEGs). Nodes represent proteins encoded by up-regulated (red) and down-regulated (white) DEGs, respectively. Node size indicates contribution made by the proteins. Edges represent protein-protein interaction. Width and transparency of edges are indicative of the network score.

STRING and Cytoscape were used to create and visualize a PPI network involving the 26 DEGs (**Figure 5**). The network comprised 18 nodes and 88 edges, with *FOS* and *IL6* each having 10 connections. An additional 16 proteins had considerable interaction with other proteins: *EGR1* (node degree=9), *FOSB* (degree=9), *CYR61* (degree=8), *MYC* (degree=8), *JUNB* (degree=7), *NR4A1* (degree=7), *NR4A2* (degree=5), *IER3* (degree=3), *EPHA2* (degree=2), *IGFBP1* (degree=2), *PPP1R15A* (degree=2), *SOCS2* (degree=2), *ADAMTS1* (degree=1), *IGFBP2* (degree=1), *PEG10* (degree=1), *PHLDA1* (degree=1). Among the top 10 identified DEGs, *PEG10*, *FOSB*, *FOS*, *IL6*, *MYC*, *JUNB*, *IGFBP2* were identified as hub genes.

### The Independent Validation Results of DEGs Expression Levels

We verified DEGs expression using RNA-seq dataset GSE126848, finding that 10 out of the 26 DEGs and five out of the top 10 DEGs found by integrated multichip analysis also exhibited significant differential expression and an identical expression trend (**Supplemental Table 4**). We then queried the 10 DEGs validated above in the PPI network, six proteins were overlapped with the discovery cohort, which consisted of 18 proteins that interacted highly with other proteins (**Supplemental Table 5**).

## Discussion

Despite the rising prevalence of nonalcoholic steatohepatitis (NASH), which is a severe form of nonalcoholic fatty liver disease (NAFLD), its underlying etiology remains unclear (21). Insights into the molecular changes that occur during NASH pathogenesis have come to light through studies that have determined the key DEGs between NASH and healthy controls (22) or by identifying DEGs among NASH, steatosis and normal tissues (23–25). However, genes expressed in both NASH and steatosis, when compared to healthy individuals, rather than differentially expressed genes, may instead drive disease progression. In the present study, we searched the Gene Expression Omnibus (GEO) database systematically for liver transcriptome studies that contained data from hepatic steatosis patients, NASH patients, and healthy subjects, identifying differentially expressed genes (DEGs) common to steatosis and NASH patients, when compared to healthy subjects. We hypothesized that such genes might play important roles in driving progression of steatosis to NASH, helping us to understand the pathogenesis of NASH more completely. Moreover, functional annotation and PPI network construction were undertaken to study the potential biological functions of DEGs.

Among the 63 DEGs identified in the comparison of steatosis patients versus healthy subjects, and the 41 DEGs identified in the NASH patients versus healthy subjects comparison, 26 DEGs were common to both comparisons, with the same pattern of up-regulation or down-regulation. Of the top-ranked 10 genes, two genes (*CYP7A1* and *PEG10*) were significantly up-regulated in both steatosis and NASH patients, while eight genes (*FOSB*, *FOS*, *IL6*, *GADD45G*, *MYC*, *SLITRK3*, *JUNB*, and *IGFBP2*) were significantly down-regulated. Among them, *PEG10*, *FOSB*, *FOS*, *IL6*, *MYC*, *JUNB*, and *IGFBP2* were identified as hub genes in the PPI network analysis.

*CYP7A1* (Cytochrome P450 family 7 subfamily A member 1) is a rate-limiting enzyme for the initial stage of bile acid biosynthesis (26), and thus acts as a cholesterol scavenger (27). Increased *CYP7A1* activity may enlarge pools of toxic bile acids, such as hydrophobic bile acids (28). Consistent with our findings, hepatic expression of *CYP7A1* is known to be significantly elevated in steatosis and NASH patients (29, 30). *PEG10* (Paternally Expressed Gene 10) is not expressed by normal livers but is over-expressed in several human cancers, such as hepatocellular carcinoma (HCC), pancreas cancer, gallbladder cancer, leukemia, breast cancer, and prostate cancer (31, 32). *PEG10* expression correlates with poor survival and increased recurrence in HCC (32). Recently, *PEG10* was found to be positively associated with disease severity in NAFLD and NASH (33).

Of the eight down-regulated genes, three (*FOS*, *FOSB*, and *JUNB*) were the transcription factor subunits of activator protein-1 (AP-1). AP-1 plays a key role in the hepatic response to acute stress, acting as a link between lipid metabolism and NAFLD (34). *FOS* proto-oncogene (also known as *c-FOS*) and its paralogue, *FOSB* proto-oncogene, were differentially expressed in various cancers and play key roles in proliferation, differentiation, migration, and the apoptosis of tumor cells (35, 36). Both *FOS* and *FOSB* were down-regulated in HCC patients (35). Little or nothing seems to be known concerning the contribution of *FOS*, *FOSB*, and *JUNB* to NAFLD etiology, necessitating further elucidation of their function. *MYC* proto-oncogene overexpression is associated with aggressive and poorly differentiated HCC (37). Mice progress spontaneously to HCC when *MYC* oncogene were activated in the liver (38). The livers of *MYC* knock-out mice developed features of NAFLD, and gradually resembled those seen in NASH (39). Our findings support the conjecture that *MYC* is consistently down-regulated in steatosis and NASH patients, *MYC* may be indispensable for the homeostasis of lipid metabolism. In our KEGG enrichment analysis, components of the MAPK signaling pathway were also significantly enriched, and aberrantly expressed MAPK genes included *FOS* and *MYC* among the DEGs. This indicates the potential role of MAPK signaling pathway in NASH progression.

*IL6* (Interleukin 6) is an inflammatory factor usually thought to be correlated with NASH severity (40, 41). Our results instead showed a clear down-regulation of *IL6* in both steatosis and NASH patients. Other studies indicate that *IL6* has beneficial effects in liver regeneration (42), anti-apoptosis (43), and the repair of liver injury (44). This suggests *IL6* is a pleiotropic cytokine and worthy of further research. *GADD45G* (Growth arrest and DNA damage 45G) functions as a stress sensor in many biological processes, inhibiting HCC development through induction of cellular senescence (45). The down-regulated expression of *GADD45G* may contribute to HCC progression (46). However, its specific role in NAFLD or NASH is not fully understood. Our findings indicate that reduced *GADD45G* expression may also be crucial in NAFLD progression. We also found the expression of *SLITRK3* (SLIT and NTRK like family member 3) was down-regulated in steatosis and NASH patients. *SLITRK3* is up-regulated in many cancers, including gastrointestinal cancer (47), and there is no clear information available about its association with NAFLD and NASH. Detailed research is still needed regarding this unsolved puzzle. We found *IGFBP2* (Insulin like growth factor binding protein 2) was down-regulated in NASH patients. Yet, adenoviral overexpression of *IGFBP2* has been shown to improve steatosis and diabetes in obese mice (48). The role of *IGFBP2* in steatosis has yet to be elucidated.

We have successfully provided insight into the functional changes that accompany the progression of NASH, yet there are limitations inherent within our study. (1) It is not known which DEGs are protective or which are disease exacerbating in the progression of NASH. (2) It remains unclear whether inferred changes in expression are causes or consequences of disease progression. (3) Experimental validation has yet to be undertaken, due to the difficulty of obtaining clinical samples from healthy and diseased livers. In the future, it will be necessary to collect liver tissue from steatosis patients, NASH patients, and healthy subjects, thus facilitating full DEG verification. In turn, this should allow the much deeper study of any potential disease-related functions exhibited by the several DEGs we have identified.

## Conclusion

In this study, we identified 26 DEGs that were present in both comparisons between NASH patients and healthy subjects and between steatosis patients and healthy subjects. Of the top-ranked 10 genes, two were significantly up-regulated (*CYP7A1* and *PEG10*) and eight were significantly down-regulated (*FOSB*, *FOS*, *IL6*, *GADD45G*, *MYC*, *SLITRK3*, *JUNB*, and *IGFBP2*). Among the identified DEGs, *PEG10*, *FOSB*, *FOS*, *IL6*, *MYC*, *JUNB*, and *IGFBP2* were identified as hub genes from our PPI network analysis. These genes may be involved in NASH progression. Once properly validated, they may provide the basis for new approaches to diagnosis or prove to be novel potential molecular targets for therapeutic intervention in NASH.

## Data Availability Statement

Publicly available datasets were analyzed in this study. This data can be found here: https://www.ncbi.nlm.nih.gov/geo/query/acc.cgi?acc=GSE48452; https://www.ncbi.nlm.nih.gov/geo/query/acc.cgi?acc=GSE63067; https://www.ncbi.nlm.nih.gov/geo/query/acc.cgi?acc=GSE89632.

## Author Contributions

JY, FB, and TZ designed the study. QW, YIL, YZ, and YAL collected the data. LC, QWu, CX, CZ, and YS analyzed and interpreted the data. JY and WY drafted the manuscript. YIL conducted an independent expression validation of DEGs. JY, YIL, FB, and TZ contributed to the critical revision of the manuscript. All authors contributed to the article and approved the submitted version.

## Funding

This work was supported by the National Natural Science Foundations of China (Grant Number: 81741059 and 81971986), the Health Department of Zhejiang Province of the People’s Republic of China (Grant Number: 2019KY098), Start-up Funding for Talent Research Program in the First Affiliated Hospital of Wenzhou Medical University (Grant Number: 2019QD011).

## Conflict of Interest

The authors declare that the research was conducted in the absence of any commercial or financial relationships that could be construed as a potential conflict of interest.
