# Supplementary figures and images for: Integrated Multichip Analysis Identifies Potential Key Genes in the Pathogenesis of Nonalcoholic Steatohepatitis
Source: Front Endocrinol (Lausanne). 2020 Nov 26;11:601745. doi: 10.3389/fendo.2020.601745 (PMC7726207; doi:10.3389/fendo.2020.601745)

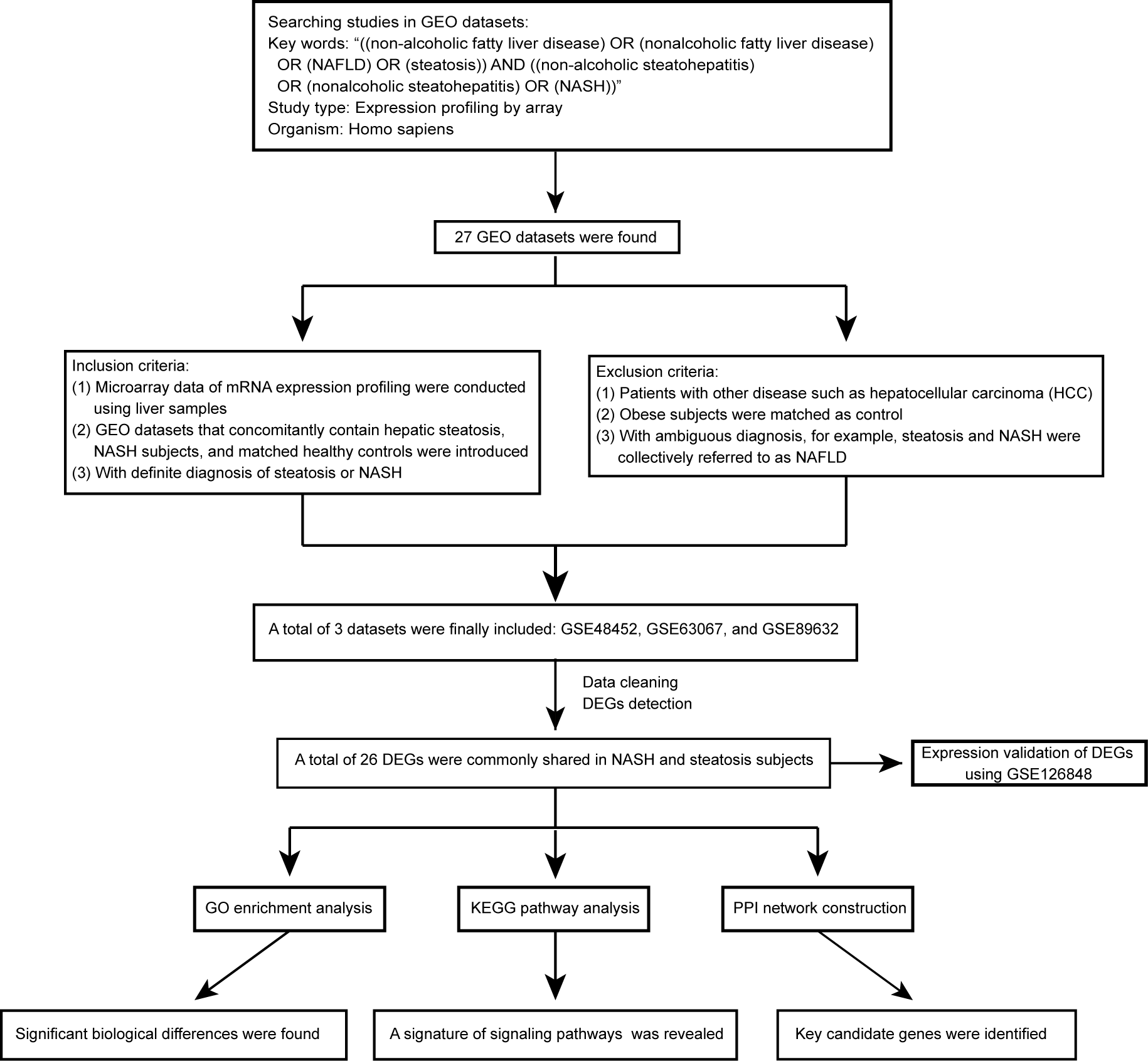

Supplement: Supplementary file 1 [file Image_1.tif]
