# Supplementary material for: Integrated Multichip Analysis Identifies Potential Key Genes in the Pathogenesis of Nonalcoholic Steatohepatitis
Source: Front Endocrinol (Lausanne). 2020 Nov 26;11:601745. doi: 10.3389/fendo.2020.601745 (PMC7726207; doi:10.3389/fendo.2020.601745)
Supplement: Supplementary file 2 [file Table_1.docx]

**TABLE S1 |** The 63 DEGs between hepatic steatosis patients and healthy subjects.

| **Gene symbol** | **Log FC** | ***P*-value** | **Adjusted *P*-value** |
| --- | --- | --- | --- |
| **Down-regulated DEGs** | | | |
| MYC | -2.06 | 2.69E-13 | 4.09E-09 |
| SOCS2 | -1.35 | 3.65E-12 | 2.14E-08 |
| THBS1 | -1.54 | 4.23E-12 | 2.14E-08 |
| P4HA1 | -1.22 | 1.37E-11 | 3.30E-08 |
| FILIP1L | -1.16 | 9.36E-11 | 1.29E-07 |
| EPHA2 | -1.20 | 1.16E-10 | 1.35E-07 |
| RRS1 | -1.19 | 1.97E-10 | 2.12E-07 |
| PHLDA1 | -1.45 | 2.10E-10 | 2.12E-07 |
| CRISPLD2 | -1.21 | 2.23E-10 | 2.12E-07 |
| IGFBP2 | -1.23 | 2.64E-10 | 2.36E-07 |
| FOSB | -2.83 | 4.58E-10 | 3.66E-07 |
| OSMR | -1.16 | 1.76E-09 | 1.02E-06 |
| ADAMTS1 | -1.60 | 1.93E-09 | 1.08E-06 |
| FAM107A | -1.13 | 4.13E-09 | 2.16E-06 |
| SLITRK3 | -1.65 | 9.24E-09 | 3.69E-06 |
| CYR61 | -1.50 | 1.76E-08 | 6.36E-06 |
| JUNB | -1.62 | 1.80E-08 | 6.36E-06 |
| GADD45G | -1.62 | 2.38E-08 | 7.86E-06 |
| KLF6 | -1.04 | 2.55E-08 | 8.09E-06 |
| IER3 | -1.35 | 5.37E-08 | 1.34E-05 |
| PPP1R15A | -1.07 | 6.20E-08 | 1.49E-05 |
| APOLD1 | -1.46 | 6.41E-08 | 1.52E-05 |
| TNFRSF12A | -1.51 | 7.30E-08 | 1.60E-05 |
| PIM1 | -1.15 | 8.52E-08 | 1.72E-05 |
| ARL14 | -1.13 | 1.01E-07 | 1.88E-05 |
| JUN | -1.09 | 1.18E-07 | 2.03E-05 |
| PNRC1 | -1.12 | 1.46E-07 | 2.38E-05 |
| FOS | -2.00 | 1.52E-07 | 2.38E-05 |
| IL6 | -1.86 | 1.88E-07 | 2.77E-05 |
| SPSB1 | -1.33 | 2.34E-07 | 3.28E-05 |
| IL1RL1 | -1.22 | 2.87E-07 | 3.75E-05 |
| CCL2 | -1.26 | 2.91E-07 | 3.75E-05 |
| EMP1 | -1.37 | 3.20E-07 | 3.90E-05 |
| KIAA0040 | -1.05 | 4.05E-07 | 4.36E-05 |
| IGFBP1 | -1.39 | 5.10E-07 | 5.13E-05 |
| FOSL2 | -1.06 | 5.95E-07 | 5.52E-05 |
| HBEGF | -1.04 | 6.36E-07 | 5.78E-05 |
| TGFB3 | -1.25 | 7.04E-07 | 6.21E-05 |
| SLC7A1 | -1.15 | 8.64E-07 | 6.97E-05 |
| SOCS3 | -1.04 | 1.05E-06 | 7.90E-05 |
| SOCS1 | -1.07 | 1.63E-06 | 1.05E-04 |
| ACTG2 | -1.05 | 1.91E-06 | 1.14E-04 |
| CCL20 | -1.38 | 2.50E-06 | 1.33E-04 |
| NR4A1 | -1.05 | 3.08E-06 | 1.54E-04 |
| RASD1 | -1.49 | 3.32E-06 | 1.62E-04 |
| FOSL1 | -1.18 | 3.55E-06 | 1.69E-04 |
| PTX3 | -1.19 | 4.71E-06 | 2.07E-04 |
| THBD | -1.03 | 5.92E-06 | 2.50E-04 |
| NR4A2 | -1.18 | 6.10E-06 | 2.54E-04 |
| PTGS2 | -1.05 | 6.38E-06 | 2.62E-04 |
| RND1 | -1.10 | 2.65E-05 | 7.18E-04 |
| EGR1 | -1.20 | 3.37E-05 | 8.40E-04 |
| SERPINE1 | -1.09 | 3.42E-05 | 8.46E-04 |
| **Up-regulated DEGs** | | | |
| CYP7A1 | 2.33 | 6.54E-10 | 4.73E-07 |
| NCAM2 | 1.38 | 1.04E-10 | 1.32E-07 |
| PEG10 | 1.20 | 8.46E-09 | 3.62E-06 |
| DBP | 1.11 | 7.51E-07 | 6.36E-05 |
| NAT8B | 1.11 | 5.22E-08 | 1.34E-05 |
| RAPGEFL1 | 1.07 | 3.73E-11 | 7.08E-08 |
| NAGS | 1.06 | 2.56E-08 | 8.09E-06 |
| CRYAA | 1.03 | 8.50E-06 | 3.27E-04 |
| RNF43 | 1.02 | 8.82E-09 | 3.62E-06 |
| NPY6R | 1.00 | 4.56E-06 | 2.04E-04 |
