# Supplementary material for: Integrated Multichip Analysis Identifies Potential Key Genes in the Pathogenesis of Nonalcoholic Steatohepatitis
Source: Front Endocrinol (Lausanne). 2020 Nov 26;11:601745. doi: 10.3389/fendo.2020.601745 (PMC7726207; doi:10.3389/fendo.2020.601745)
Supplement: Supplementary file 3 [file Table_2.docx]

**TABLE S2 |** The 41 DEGs between NASH patients and healthy subjects.

| **Gene symbol** | **Log FC** | ***P*-value** | **Adjusted *P*-value** |
| --- | --- | --- | --- |
| **Down-regulated DEGs** | | | |
| FOSB | -3.19 | 3.43E-15 | 1.30E-11 |
| FOS | -2.20 | 6.26E-11 | 3.80E-08 |
| IL6 | -1.72 | 2.71E-09 | 7.47E-07 |
| GADD45G | -1.71 | 4.52E-13 | 5.71E-10 |
| MYC | -1.64 | 1.11E-11 | 8.45E-09 |
| SLITRK3 | -1.58 | 3.22E-08 | 4.40E-06 |
| JUNB | -1.58 | 9.20E-11 | 4.65E-08 |
| IGFBP2 | -1.47 | 4.17E-13 | 5.71E-10 |
| SOCS2 | -1.43 | 8.59E-14 | 1.86E-10 |
| NR4A2 | -1.40 | 9.10E-09 | 1.92E-06 |
| APOLD1 | -1.28 | 3.44E-08 | 4.53E-06 |
| ADAMTS1 | -1.27 | 1.13E-07 | 1.06E-05 |
| IGFBP1 | -1.26 | 4.58E-08 | 5.50E-06 |
| P4HA1 | -1.22 | 8.25E-16 | 4.17E-12 |
| NR4A1 | -1.20 | 1.74E-09 | 5.50E-07 |
| RASD1 | -1.19 | 3.92E-06 | 1.71E-04 |
| CYR61 | -1.18 | 3.63E-08 | 4.71E-06 |
| RND1 | -1.13 | 9.40E-07 | 5.68E-05 |
| EGR1 | -1.10 | 2.36E-05 | 6.67E-04 |
| ACTG2 | -1.08 | 1.92E-07 | 1.65E-05 |
| PPP1R15A | -1.07 | 2.48E-12 | 2.60E-09 |
| SIK1 | -1.04 | 1.27E-08 | 2.44E-06 |
| EPHA2 | -1.03 | 9.48E-10 | 3.42E-07 |
| PHLDA1 | -1.03 | 1.82E-07 | 1.58E-05 |
| IER3 | -1.02 | 4.10E-07 | 2.89E-05 |
| ZFP36 | -1.01 | 5.57E-08 | 6.30E-06 |
| GADD45B | -1.00 | 9.91E-12 | 7.91E-09 |
| **Up-regulated DEGs** | | | |
| CYP7A1 | 2.31 | 2.87E-11 | 1.89E-08 |
| AKR1B10 | 2.22 | 1.13E-09 | 3.90E-07 |
| PEG10 | 1.80 | 5.42E-16 | 4.11E-12 |
| FMO1 | 1.60 | 7.75E-17 | 1.18E-12 |
| LGALS4 | 1.20 | 4.61E-10 | 1.75E-07 |
| TMEM154 | 1.16 | 4.91E-12 | 4.38E-09 |
| GINS2 | 1.15 | 4.89E-11 | 3.09E-08 |
| EEF1A2 | 1.11 | 2.19E-07 | 1.83E-05 |
| CXCL9 | 1.11 | 6.51E-08 | 7.02E-06 |
| FADS2 | 1.10 | 4.39E-08 | 5.33E-06 |
| FABP4 | 1.07 | 2.52E-09 | 7.35E-07 |
| ME1 | 1.05 | 1.27E-13 | 2.15E-10 |
| MEP1B | 1.04 | 3.34E-09 | 8.94E-07 |
| TRHDE | 1.02 | 7.74E-11 | 4.20E-08 |
