# Supplementary material for: Integrated Multichip Analysis Identifies Potential Key Genes in the Pathogenesis of Nonalcoholic Steatohepatitis
Source: Front Endocrinol (Lausanne). 2020 Nov 26;11:601745. doi: 10.3389/fendo.2020.601745 (PMC7726207; doi:10.3389/fendo.2020.601745)
Supplement: Supplementary file 5 [file Table_4.docx]

**TABLE S4 |** Validation of DEGs using dataset GSE126848.

| **Gene symbol** | ***P*-value** | | |
| --- | --- | --- | --- |
|  | **Steatosis vs. Healthy control** | **NASH vs. Healthy control** | |
| **Down-regulated DEGs** | | |  |
| P4HA1 | 6.24E-17 | 1.53E-31 | |
| EGR1 | 6.48E-14 | 4.56E-12 | |
| IGFBP2 | 1.22E-09 | 4.32E-13 | |
| SLITRK3 | 4.19E-03 | 1.38E-05 | |
| SOCS2 | 2.33E-08 | 2.59E-06 | |
| IGFBP1 | 5.23E-03 | 9.05E-03 | |
| FOS | 5.40E-04 | 1.57E-02 | |
| EPHA2 | 1.45E-02 | 2.79E-04 | |
| **Up-regulated DEGs** | | |  |
| PEG10 | 8.23E-06 | 6.26E-10 | |
| CYP7A1 | 2.80E-02 | 1.11E-05 | |
