# Supplementary material for: Integrated Multichip Analysis Identifies Potential Key Genes in the Pathogenesis of Nonalcoholic Steatohepatitis
Source: Front Endocrinol (Lausanne). 2020 Nov 26;11:601745. doi: 10.3389/fendo.2020.601745 (PMC7726207; doi:10.3389/fendo.2020.601745)
Supplement: Supplementary file 6 [file Table_5.docx]

**TABLE S5 |** PPI network validation using dataset GSE126848.

| **Gene symbol** | **Degree of interaction** |
| --- | --- |
| FOS | 45 |
| EGR1 | 32 |
| IGFBP1 | 17 |
| SOCS2 | 10 |
| PEG10 | 7 |
| SLITRK3 | 4 |
| P4HA1 | 3 |
| IGFBP2 | 2 |
